# Mr. Scott's Case of Hydrothorax

**Published:** 1809-06

**Authors:** James Scott

**Affiliations:** Surgeon. H. M. S. Euryalus, Sheerness


### To the Editors of the Medical and Physical Journal.

GENTLEMEN,

THE diagnostic part of medical science is universally allowed to be of the highest importance, and much labor and patient investigation have been bestowed on it. In the present age, philosophers are not so apt, as formerly, to follow the opinion of the closet writer, but have endeavoured, by closely observing the operations of Nature in actual disease, to gain that knowledge which can only be acquired at the patient's bed-side.

Much, however, as practical acquirement is valued, it cannot be denied that the symptoms of many diseases are so differently marked by modern writers, that the young practitioner, who, relying on what he may conceive the highest authority, shall be called to a patient, and will often look in vain for those which have been said to ascertain the complaint.

This want or imperfection of diagnostics, was particularly striking in a Case of Hydrothorax, the history and fatal termination of which I shall proceed to state.

ROBERT ALDRIDGE, seaman, ætat. 28, of a spare habit, and who had been much less addicted to drinking than sailors generally are, was attacked with severe pain all over the abdomen, with slight inclination to vomit. At times he had violent spasmodic twitchings of the bowels, and other symptoms which indicated the presence of worms in the intestinal canal. With a view to their expulsion, he took gr. vj. of calomel and xv of jalap, and a short time after voided a worm of the teres kind, nearly 14 inches long, which came away alive. The pain was immediately removed, but much irritation of the stomach remained, and an anodyne oleaginous draught was given with the wished for effect. This was on the 11th of April, 1808.

On the following day, he complained of a slight catarrhal affection, which was treated with demulcent diluents, syrup of squills in small doses, and an anodyne at bed-time, by which means he was recovering rapidly until the 15th, when having incautiously exposed himself to cold, symptoms of pneumonia supervened in a high degree, for which he was bled in the evening to fourteen ounces, and took an antimonial.

On the 16th, in the morning, the symptoms were very little little relieved, and the bleeding was repeated to twenty ounces; a blister was applied to the thorax and fomentations to the belly and chest; he was ordered barley water and infusion of linseed for common drink; eight ounces of blood were taken away in the evening.

On the 17th, he was quite easy and coughed only occasionally. The expectoration was, however, rather difficult, on which account I again had recourse to the squill-mixture and anodynes, with the best effect.

On the 18th, he complained of a sensation of "soreness of the bowels;" pulse frequent and small; tongue parched; some thirst; almost constant, yet gentle vomiting, and other symptoms indicative of Enteritis Erysipelatosa.

The abdomen was fomented; he took frequently a little infusion of toasted oat-meal cake, which was eminently successful in allaying the vomiting; and when that ceased, $\frac{1}{6}$ of a grain of antimon. tartar. every two hours: emollient clysters were also administered; and on the 19th of April, 1808, as the ship was going to sea, I sent him to the Royal Naval Hospital at Halifax, with scarcely any apparent complaint but debility.

In August, 1808, we retured to Halifax, and I visited him at the hospital, the comforts of which seemed in some degree to have restored him, for he had taken little or no medicine: considerable weakness, however, remained, when I received him on board on the 7th of Sept. 1808.— From that time till the 15th of the same month, he appeared to be recovering his appetite and strength; was much improved in appearance; and slept soundly. The favourable symptoms contined till the morning of the 15th, when he complained of irregular chills, great prostration of strength, and "cloudiness" of his eyes, as he expressed himself. He also mentioned a pain in the region of the liver, and had an unpleasant, dry, tickling cough; but pressure on the hypochondrium caused no increase of pain. As the complaint appeared to proceed from inactivity of the liver, external friction was employed, and a cordial carminative given, which procured him almost immediate relief. In the evening he felt quite easy, and an opiate was given.

On the 16th, the cold fits recurred in the morning, but yielded to a cordial draught. His countenance, however, continued very pale; he had an irregular hurried pulse, and appeared to be verging fast to dissolution, which took place at seven o'clock in the evening.

### Appearances on Dissection.

The brain was carefully examined, and exhibited no marks of disease. The thorax contained near a gallon of serous fluid, and an unusual quantity was also found in the pericardium. The right auricle appeared to be much above the common size; and just before the entrance of the pulmonic artery, below the semilunar valves, the rim of the right ventricle was quite cartilaginous, and had a rough ridge on it, completely ossified, of a gritty feel, and not unlike the extremity of a joint whose cartilage has been abraded by exposure to the weather.

There was not any coagulated blood in either of the ventricles. The fluids, both in the thorax and pericardium were of a deep yellowish colour. The left auricle was rather smaller than usual, or perhaps the great size of the right one made it appear so. The lungs were very sound, but both lobes adhered to the pleura in several places. The stomach did not appear to be at all diseased, but the liver was scirrhous, and a good deal enlarged. The gall-bladder was nearly empty, and the little gall it contained appeared very dark coloured and viscid. The spleen seemed very small and hard. The omentum was almost entirely deprived of its fat, and had a yellowish tinge in several places. The kidneys were very healthy, but had a smaller portion of fat than usual; the capsulæ renales appeared sound, but were invested with a sort of cellular substance only, of a yellow hue. The legs and thighs had not suffered much emaciation. From this statement of appearances, it is evident that effusion of fluid into the cavities of the thorax and pericardium was the immediate cause of his death, and the only question is, when did the effusion take place? It does not appear probable, that any fluid existed long before his death, because the usual symptoms of hydrothorax were not present till about an hour previous to his dissolution; and yet it seems difficult to admit that so great a quantity of serum was poured out in so short a time. But if it was not produced in that sudden manner, how happened it that the patient endured a recumbent posture without uneasiness? and could he, with his chest full of water, have enjoyed sound and uninterrupted sleep? Whence also proceeded the bilious tinge of the fluid? And although no anasarca prevailed, did not the accumulation of bilious serum in the thorax and pericardium indicate a disposition to general dropsy, which we know often follows a diseased liver, probably in consequence of debility, in-

 duced duced by imperfect digestion, from the small quantity or improper quality of the bile, rather than the supposed mechanical effect of the enlarged liver on the vena cava? About a quarter of an hour before the patient died, he had a fainting fit, from which he recovered with much difficulty, and almost immediately relapsed into a state of insensibility, in which his existence terminated. Did not the effusion take place during the first syncope? Or what shall we consider truly characteristic of this disease? The pulse neither intermitted nor was irregular, till the day of his death. There was no difficulty of respiration nor starting from sleep. No remarkable paleness of countenance till towards the close of the disease. No great thirst, nor scarcity of urine, and very little emaciation. I ask these questions for the sake of information; I hope some of your readers will think them worthy of consideration, and should they be acquainted with any symptoms which would more clearly point out the existence of hydrothorax, than those mentioned by authors, I trust they will be pleased to make them publicly known for the benefit of mankind.

I am, &c.

JAMES SCOTT, Surgeon.

H. M. S. Euryalus, *Sheerness, May* 13, 1809.

*** We must request Mr. Scott, whilst we thank him for his useful communication, to inform us how long after death the body was opened, and whether there was any reason to suspect transudation after death? Ed.

